# Disease of the Lungs

**Published:** 1901-03-23

**Authors:** 


### Disease of the Lungs.

(*Continued from page* 423.)

Holcomb[8] gives a report upon the treatment of tuberculosis by means of tuberculocidin, which consists of Koch's tuberculin from which has been removed the poisonous properties. This has been accomplished by Professor Klebs, by means of sodium bismuth iodide added to the filtered cultures. The filtrate is then treated with absolute alcohol which precipitates an albumose soluble in water. This in solution is tuberculocidin. He states that tuberculocidin may be used for all forms of tuberculosis in which the toxic effects of the virus have not markedly developed. The dose used is 1½ ♏ of a 1 per cent. solution increasing daily as tolerated until ♏xxx. is attained. Time required for treatment is from two to four months, and in this time from 100c.c. to 200c.c. will be needed. Some patients require a repetition of the treatment for some years. The material is administered hyperdermically, but it can be given by rectal injections. He gives the notes of one case and tells of seven others, where well-established phthisis was cured by this treatment; in the case quoted bacilli were absent with the other signs for four years. He states that it is useless in rapid consumption. Tropon[9] is recommended as a nutritious form of administering albumen in cases of phthisis by Knopt. He states that it rarely causes digestive disturbances, and that it can be taken for long periods of time without aversion, and that it is excessively cheap. Night sweats of phthisis may be checked by the following lotion :—

| | |
|---|---|
| ℞ Balsam of Peru . . | 1 pt. |
| Formic Acid . . . | 5 pts. |
| Chloral Hydrate . . | 5 pts. |
| Trichloracetic Ac. . . | 1 pt. |
| Absolute Alcohol . . | 100 pts. |

Opinions[10] differ as to the value of the anti-tubercular serums. McFarland is of the opinion that they are all deleterious. Boardman Reed considers they are useful in diagnosis. Whittaker agrees with him, and Denison thinks that they are a "potent influence for good." Karl von Ruck thinks the toxins of tubercle bacilli have an undoubted favourable influence on tubercular processes.

Solly[11] has analysed 8,000 cases treated by climatic change, and comes to the conclusion that inland is better than sea air, dryness is better than dampness, and high climates better than low. A good case of the "prompt abatement and arrest of symptoms (of tuberculosis of the lungs) tending at one time to indicate a rapid progress towards a fatal termination" is given by Little and Ross.[12] The patient had been to South Africa for two years, but came back much worse. After open-air treatment in England his condition was immensely improved in a few months. The symptoms at first were those of rapid wasting, hoarse husky voice, bronchorrhoeal sputum crowded with T. bacilli. Both apices were involved, with a vomica in left, ulcerated larynx, and a temperature of 101° to 102° daily. Copious night-sweats and slight anasarca, weight equal 9 stone. The result is—weight has risen to 10 stone, his voice has lost nearly all huskiness, and beyond slight signs at the apices there are no other symptoms of disease.

Stretton[13] gives a case of tuberculous disease of the apex of right lung, removed by operation, with recovery. The operation was conducted by means of a vertical incision through the third rib. The diseased lung was adherent and separated with difficulty, but was at last surrounded by means of a serre-nœud and cut away. The hæmorrhage was considerable and persistent, but later the wound healed soundly. The author states that he would use a transverse incision on the next occasion.

Experiments[14] have been made in Adirondack sanatorium to ascertain how many patients suffering from tuberculosis carried the tubercle bacillus on their hands. It was found that only two out of ten were free from the bacilli.

Swithinbank[15] urges the importance of dust infected by tuberculosis. He quotes the report of the French Government Commission where it is stated by Professor Brouardel that of all post-mortems on the bodies at the Morgue, dead by other means than tuberculosis, one half of those over thirty years of age show cicatrised tubercles in the lungs.

Heller has estimated that 7,200,000,000 bacilli are expectorated daily by the phthisical patient. These frequently are thrown into the public ways, and on being desiccated to powder are a terrible danger to all who breathe them. Experiments by Koch, Cervet, and others have proved how infective dried sputum is. Even on a sunny grass plot sputum was infective after fourteen days.

Railway stations in particular, being shaded from sun, swept by drying winds and frequently dusted by large soft brooms which raise much of it into the air, are hotbeds of the infection.

[8] Med. Times, Jan. 1, 1901. [9] Practitioner, Dec. [10] Quar. Med. Jour., Nov. [11] Quar. Med. Jour., Nov. [12] Lancet, Dec. 1. [13] Lancet, Dec. 8. [14] Practitioner, Dec. [15] Tuberculosis, Jan. 1901.

